# Complete genome sequence of *Limosilactobacillus reuteri* strain iVE-9, isolated from the fecal sample of a healthy human

**DOI:** 10.1128/mra.01134-25

**Published:** 2025-11-24

**Authors:** Mallory J. Van Haute, Katherine Chacón-Vargas, Shara R. P. Yumul, Chloe M. Christensen, Robert Hutkins, Thomas A. Auchtung

**Affiliations:** 1 Synbiotic Health, Inc, Lincoln, Nebraska, USA; 2 Department of Food Science and Technology, University of Nebraska-Lincoln 14719, Lincoln, Nebraska, USA; 3 Nebraska Food for Health Center, University of Nebraska-Lincoln 14719, Lincoln, Nebraska, USA; Wellesley College, Wellesley, Massachusetts, USA

**Keywords:** *Limosilactobacillus reuteri*, iVE-9, exopolysaccharides, B vitamins, bacteriocin, indole-3-lactic acid (ILA), gamma-aminobutyric acid (GABA)

## Abstract

*Limosilactobacillus reuteri* inhabits the gastrointestinal tract of a variety of hosts. Here, we present the complete genome sequence of a human isolate, *L. reuteri* iVE-9, that encodes for several potentially beneficial metabolic traits, including production of exopolysaccharides, B vitamins, a bacteriocin, indole-3-lactic acid, and gamma-aminobutyric acid.

## ANNOUNCEMENT

*Limosilactobacillus reuteri* has been isolated from humans, a variety of animals (1–3), and fermented foods (4–6). *L. reuteri* has been widely studied to understand host adaptation and diversification into host-specific lineages (1). Clinical trials with *L. reuteri* have shown health-promoting benefits related to immunity (7) and a range of gastrointestinal diseases (8). In addition, diverse desirable properties have been described for specific strains, including the production of antimicrobial compounds (9, 10), B vitamins (11), immunomodulatory factors (12), and exopolysaccharides (13).

*L. reuteri* iVE-9 was obtained from a healthy woman living in Nebraska (USA). A donated fecal sample was used to inoculate 37°C anoxic fermentation media (14) (pH 7.0) containing the disaccharide palatinose as the only carbohydrate. The mixture was grown overnight, then successively transferred every 24 h in the same medium (15). After 96 h, the strain was subsequently isolated on agar plates of MRS (BD Difco recipe) with palatinose as the only carbohydrate and identified by Sanger sequencing of the 16S rRNA gene using primers Bact8F (AGAGTTTGATCCTGGCTCAG) and Bact1492R (GGYTACCTTGTTACGACTT) (16).

For DNA isolation, *L. reuteri* iVE-9 was grown overnight to late exponential phase in standard MRS broth at 37°C under anaerobic conditions. The culture was pelleted by centrifugation and sent to CD Genomics (Shirley, NY, USA). Total genomic DNA was extracted using a DNeasy UltraClean Microbial kit (Qiagen) following the manufacturer’s instructions. DNA was sheared into 10 kb fragments for SMRTbell (v.3.0) library creation using a gTUBE (Covaris) and sequenced on a PacBio Revio (v.2 chemistry). SMRT Link 8.0 software was used for data processing.

A total of 146,905 reads (mean 19,713 bp; N_50_ = 19,873 bp) were corrected and trimmed with Canu (v.2.1.1) and assembled into a single circular contig (2,161,134 bp; 38.89% GC) with Flye (v.2.9.1) using default parameters. Annotation performed by PGAP (v.6.10) (17) identified 2,131 protein-coding genes, 73 tRNAs, 18 rRNAs, and 1 tmRNA. Additional annotation was performed using Prokka (v.1.14.6) (18), eggNOG-mapper (v.2) (19), and BAGEL4 (20). dbCAN3 (21) identified 26 carbohydrate gene clusters for glycoside hydrolase families with functions including alpha-glucan, starch, and chitin metabolism. iVE-9 is phylogenetically basal to *L. reuteri* lineage II (containing human and herbivore isolates) by maximum likelihood analysis (FastTree v.2.1) (22) of 846 concatenated core genes compiled by Roary (v.3.13) (23). Default parameters were used for all software.

**Fig 1:**
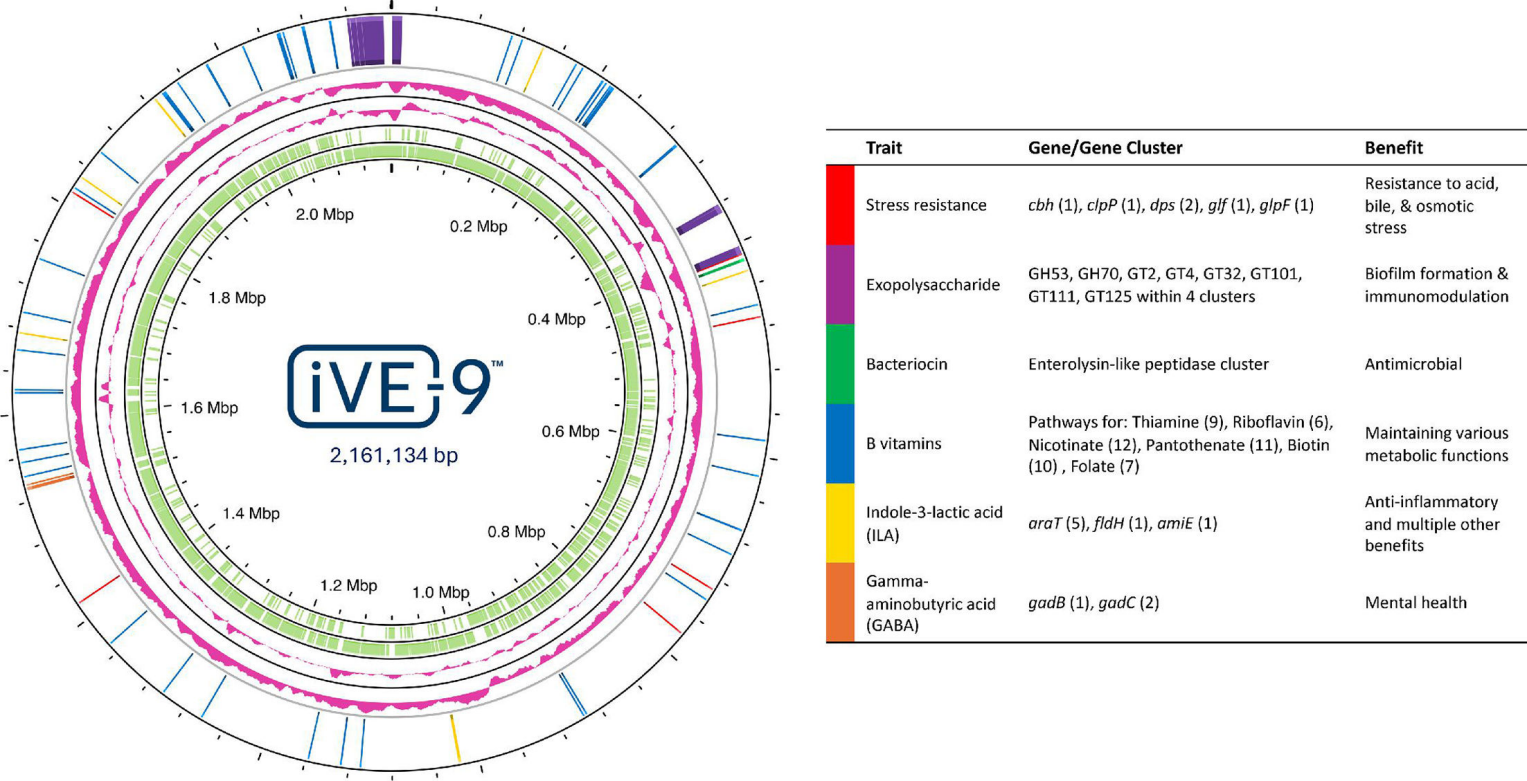
Genome map of *Limosilactobacillus reuteri* iVE-9: from the inner to outer circle are tick marks (Mbp), CDS reverse strand (light green), CDS forward strand (light green), GC content (pink), GC skew (pink), and genes of probiotic interest (stress resistance, red; exopolysaccharide, purple; bacteriocin, green; B vitamins, blue; indole-3-lactic acid, yellow; gamma-aminobutyric acid production, orange). The figure was generated with Proksee (32).

Traits for which iVE-9 may provide host advantages were examined (Fig. 1). No antibiotic resistance or virulence genes were detected (CARD RGI [24] and VFDB [25]). Genes were identified that are predicted to be involved in stress resistance (26), the metabolism of tryptophan to the anti-inflammatory compound indole-3-lactic acid (27), and the production and transport of the neurotransmitter gamma-aminobutyric acid (28), which may confer mental health benefits. The genome contains putative gene clusters involved in exopolysaccharide synthesis (29) and production of an antimicrobial bacteriocin (30). Additionally, using the KEGG database, we identified pathways for synthesis of B vitamins (31). In summary, the genome of *L. reuteri* iVE-9 suggests it has potential probiotic properties that may benefit human health.

## Data Availability

The iVE-9 BioProject, BioSample, SRA, and GenBank complete assembled genome accession numbers are PRJNA1270077, SAMN48813938, SRR33773734, and CP194781.
